# Induction of Multiple miR-200/182 Members in the Brains of Mice Are Associated with Acute Herpes Simplex Virus 1 Encephalitis

**DOI:** 10.1371/journal.pone.0169081

**Published:** 2017-01-03

**Authors:** Anna Majer, Kyle A. Caligiuri, Kamilla K. Gale, Yulian Niu, Clark S. Phillipson, Timothy F. Booth, Stephanie A. Booth

**Affiliations:** 1 Department of Medical Microbiology and Infectious Diseases, College of Medicine, Faculty of Health Sciences, University of Manitoba, Winnipeg, Manitoba, Canada; 2 Molecular PathoBiology, National Microbiology Laboratory, Canadian Science Centre for Human and Animal Health, Public Health Agency of Canada, Winnipeg, Manitoba, Canada; 3 Viral Diseases, National Microbiology Laboratory, Canadian Science Centre for Human and Animal Health, Public Health Agency of Canada, Winnipeg, Manitoba, Canada; Rowan University, UNITED STATES

## Abstract

Important roles of microRNAs (miRNAs) in regulating the host response during viral infection have begun to be defined. However, little is known about the functional roles of miRNAs within an *in vivo* acute viral encephalitis model. We therefore identified global changes in miRNA expression during acute herpes simplex virus type 1 (HSV-1) encephalitis (HSVE) in mice. We found that many of the highly upregulated miRNAs (miR-155, miR-146a and miR-15b) detected in HSV-1 infected brain tissue are known regulators of inflammation and innate immunity. We also observed upregulation of 7 members belonging to the related group of miRNAs, the miR-200 family and miR-182 cluster (miR-200/182). Using *in situ* hybridization, we found that these miRNAs co-localized to regions of the brain with severe HSVE-related pathology and were upregulated in various cell types including neurons. Induction was apparent but not limited to cells in which HSV-1 was detected by immunohistochemistry, suggesting possible roles of these miRNAs in the host response to viral-induced tissue damage. Bioinformatic prediction combined with gene expression profiling revealed that the induced miR-200/182 members could regulate the biosynthesis of heparan sulfate proteoglycans. Using luciferase assays, we found that miR-96, miR-141, miR-183 and miR-200c all potentially targeted the syndecan-2 gene (*Sdc2*), which codes for a cell surface heparan sulfate proteoglycan involved in HSV-1 cellular attachment and entry.

## Introduction

Herpes simplex virus type 1 (HSV-1) typically infects the host at the epithelial surface through mucosal secretions, becomes latent and resides within the trigeminal ganglia. Periodically, the virus reactivates and causes recurrent lesions at primary sites of infection. Rarely does the virus reactivate and disseminate within the brain tissue causing herpes simplex encephalitis (HSVE) [1]. Nevertheless, HSVE is the most commonly recognized cause of acute encephalitis with an incidence rate of 2–4 individuals/million annually [2]. If untreated, mortality rates of HSVE can reach ≥70% and even after successful treatment the severe neurological damage that occurs during disease can result in lifelong disability [3]. Although a vast array of data has been collected over the years describing the gene networks that are disrupted during HSVE [4–6], the upstream regulatory pathways that control the expression of these genes within an *in vivo* environment remain poorly understood.

MicroRNAs (miRNAs) are an abundant class of short, non-coding RNAs that regulate gene expression at the post-transcriptional level [7]. By affecting gene regulation, miRNAs are therefore intricately involved in a wide range of diverse biological pathways including inflammatory responses [8] as well as pathological conditions such as viral infections (reviewed in [9–11]). Due to their widespread regulatory roles, it is not surprising that numerous viruses use miRNAs to regulate aspects of their life-cycle. Some viruses, including members of the *Herpesviridae* family, encode miRNAs that likely regulate their own viral gene expression [12]. Additionally, modulation of cellular miRNAs have been observed in numerous viruses such as HSV-1, hepatitis C virus (HCV), human cytomegalovirus (HCMV), human immunodeficiency virus (HIV) and West Nile virus (WNV) [13–21]. However, little is known about how these dysregulated cellular miRNAs affect virus-host interactions and disease pathogenesis, although the number of reports which describe the critical roles for some miRNAs in viral replication and spread [14, 22–27] have increased in the past several years. For HSV-1, recent data has shown that a neuronal-enriched miRNA (miR-138) targets *ICP0*, which encodes a viral protein that functions to reactivate the virus from latency [28]. As a result, expression of miR-138 promotes viral latency within neurons [29]. MiRNAs can also have an indirect effect on HSV-1 pathogenicity. MiR-155 deficient mice are highly susceptible to HSV-1 replication, resulting in enhanced mortality [30]. These data highlight the critical roles miRNAs play during viral pathogenicity. However, the role of cellular miRNAs within an *in vivo* HSV-1 infection model which causes acute herpes simplex encephalitis remain largely unexplored and warrants further study.

To address this gap in knowledge, we identify global changes in miRNA expression during HSV-1 encephalitis using next generation sequencing (NGS). We validated deregulation of several miRNAs and noted that many of the highly upregulated (miR-155, miR-146a and miR-15b) are regulators of inflammation and innate immunity. We also observed the upregulation of 7 miRNAs belonging to the related and often co-transcribed miRNA-200 family (miR-200a,b,c/miR-141/miR-429) and miRNA-182 cluster (miR-182/miR-183), henceforth collectively referred to as miR-200/182. We found that several of these miRNAs were significantly induced in areas containing HSV-1 positive cells. Using *in situ* hybridization (ISH), we found that miR-141, miR-200a and miR-183 expression was induced in cells that appeared to include not just myeloid cells but also other resident brain cells such as neurons and endothelial cells. Bioinformatic analysis combined with gene expression data showed that these miRNAs have numerous putative targets that were also downregulated during HSVE. Some of these miRNA gene targets are involved in neuronal development and the biosynthesis of heparan sulfate proteoglycans (HSPGs). Several members of HSPGs, such as syndecan 2 (SDC2) are important for HSV-1 entry into the cell [31]. Using luciferase assays, we found that miR-96, miR-141, miR-183 and miR-200c all downregulated the expression of *Sdc2*. Overall, our data suggests that miR-200/182 induction may result in downregulation of *Sdc2* in an *in vivo* mouse model of HSVE.

## Materials and Methods

### Ethics statement

All procedures involving live animals were approved by the Canadian Science Centre for Human and Animal Health—Animal Care Committee (CSCHAH-ACC) according to the guidelines set by the Canadian Council on Animal Care. All protocols were designed to minimize animal suffering. The approval identification for this study was the animal use document (AUD) #H-13-015 and the amendment to the AUD #H-06-003.

### HSV-1 propagation and infection of mice

HSV-1 strain F was propagated and titers determined by standard plaque assay using Vero cells (African green monkey kidney, ATCC CCL-81). Adult female SJL/J mice were purchased from the Charles River Laboratories (Charles River, MA) and were intracerebrally inoculated into the left cerebellum with 10 μL of 1 x 10^6^ plaque forming units (PFU) of HSV-1 strain F following anesthesia by injection of 20 mg/kg of xylazine and 75 mg/kg of ketamine. Equal numbers of control mice were mock-infected by inoculation with PBS or Vero cell suspension. Experimental groups consisted of 8 mice per treatment; 4 mice were used for RNA extraction and 4 for histological analysis from each group. The Vero cell suspension served as a control to confirm that animals did not mount an immune response to the cellular debris that can arise during the harvesting of the virus. Animals were checked twice daily to monitor health following infection, and were housed together based on the administered treatment with plenty of food and water during the study. Mice were sacrificed by cervical dislocation once they developed clinical symptoms of HSVE (28 or 48 hours post infection). A combination of apathy, ruffled fur and hunched posture was used as endpoint criteria. However, due to the rapid progression of disease some mice exhibited more severe clinical signs prior to sacrifice, such as seizures. Brains were removed at sacrifice and processed by either fixing in 10% neutral buffered formalin-fixed and paraffin-embedded (FFPE) for pathology, or placed in optimal cutting temperature medium for RNA extraction.

### Histology

To determine gross histopathological changes, FFPE brain tissue was sectioned into 5 μm thick sagittal sections, placed on Superfrost microscope slides (Fisher) and backed at 37°C overnight. Sections were deparaffinized using two changes of xylene and rehydrated by immersing in 100%, 90% and then 70% ethanol. Sections were stained for nuclear structures using Harris hematoxylin (Surgipath) for 2 minutes followed by differentiation in 1% acid alcohol (Surgipath) and treatment with Scott’s tap water for 2 minutes. Subsequently, sections were counterstained for cytoplasmic structures using eosin (Surgipath) for 2 minutes. Slides were dehydrated with 70%, 90% and 100% ethanol, cleared in xylene and mounted using Permount (Fisher Scientific).

### Immunohistochemistry

To detect HSV-1 positive cells, sections were processed as described for histopathology up to and including the ethanol rehydration step. Subsequently, endogenous peroxidase activity was blocked using 3% hydrogen peroxide (H_2_O_2_) for 10 minutes, rinsed in water and antigen retrieval was performed using 10 mM Sodium Citrate buffer (pH 6.0) at 120°C for 10 minutes. Slides were rinsed in water and incubated with primary antibody at 4°C overnight. Slides were rinsed in TBS/T and treated with rabbit antibody amplifier for 15 minutes using the MaxPolyTwo^™^ Polymer HRP rabbit detection kit (Max Vision Biosciences). To resolve HSV-1 positive cells, DAB (Max Vision Biosciences) was incubated with the slides for 1 minute. To counterstain, slides were rinsed in water, stained with Gill III hematoxylin (Surgipath) for 10 seconds, rinsed in water, treated for 2 minutes in Scott’s tap water (Surgipath), rinsed again in water, air dried overnight, clarified in xylene and mounted using Permount.

To co-localize HSV-1 positive cells with either microglia or astrocytes, sections following DAB incubation to detect HSV-1 positive cells (described above) were treated as follows. Sections only designated to detect microglia were treated with 3% H_2_O_2_ for 10 minutes to block endogenous peroxidase activity. All slides were then rinsed in water and blocked using 5% normal rabbit serum (Dako) diluted in EnVision FLEX antibody diluent for 10 minutes followed by the Max Homo rabbit blocking reagent (Max Vision Biosciences) for 1 hour. Either anti-IBA1 or anti-GFAP was incubated with the slides for 1 hour at room temperature at the designated dilutions to stain for microglia or astrocytes, respectively. Subsequently, sections designated for IBA1 visualization were incubated with the polymer HRP anti-rabbit secondary antibody (Max Vision Biosciences) for 15 minutes followed by the substrate Vina Green (Inter medical) for 10 minutes at room temperature. Conversely, slides designated for astrocyte visualization were incubated with polymer AP anti-rabbit secondary antibody (Max Vision Biosciences) for 15 minutes followed by the substrate Fast Red (Inter medical) for 10 minutes. Section were counterstained, clarified and mounted as described above.

Antibodies used: anti-HSV-1 (rabbit; 1:20000; Dako); anti-GFAP to detect astrocytes (rabbit; 1:4000; Dako) and anti-IBA1 to detect microglia (rabbit; 1:2000; Wake). All antibodies were diluted in EnVision FLEX antibody diluent (Dako).

### Immunofluorescence

Each brain sample was processed as described in the immunohistochemistry procedure up to and including the antigen retrieval step. Sections were then blocked in 1:20 normal goat serum (Cedarlane) that was diluted in EnVision FLEX antibody diluent for 1 hour at room temperature. Slides were incubated with primary antibody at 4°C overnight. Sections were rinsed twice with TBS/T and incubated for 1 hour at room temperature with secondary antibody. Slides were rinsed with distilled water and nuclei were counterstained with DAPI (Life Technologies Inc.) at a 1:1000 dilution for 20 minutes at room temperature. Slides were rinsed with distilled water, air dried overnight and mounted using Permount. Antibodies used: anti-HSV-1 (rabbit; 1:1000; Dako); anti-rabbit secondary conjugated to Alexa Fluor 594 (goat; 1:1000; Life Technologies).

### Next generation sequencing sample preparation and analysis

Total RNA was extracted from ½ brain (~0.25g of tissue) using *mir*Vana miRNA Isolation Kit (Life Technologies) following the manufacturer’s instructions. RNA concentration and quality was assessed for each sample in duplicate via the 2100 Bioanalyzer RNA 6000 Nano Kit (Agilent Technologies Inc.). Approximately 2 μg of total RNA exhibiting RNA integrity number quality greater than or equal to 7.5 were shipped to The Centre for Applied Genomics at the Hospital for Sick Children (Toronto, Ontario, Canada) where the next generation sequencing (NGS) library and sequencing reaction were performed. Briefly, the Illumina Small RNA Sample Preparation Kit (San Diego, CA) was used to isolate small RNAs, ligate adaptors onto the samples and generate a cDNA library following manufacturer’s recommendations (Illumina 2007, San Diego, CA). The cDNA library was ligated to the flow cell and the non-template strands were cleaved off by using an Illumina Genome Analyzer Cluster Station via the Cluster Generation Kit following manufacturer’s specifications (Illumina 2008, San Diego, CA). Samples were run in the Illumina Genome Analyzer II (Illumina, San Diego, CA) using the two 36-cycle Sequencing kit (Illumina, San Diego, CA) following the manufacturer’s protocol (Illumina 2008) to obtain a single-read, 72 base pair next generation sequencing run on an Illumina Genome Analyzer II (San Diego, CA).

Primary analysis was performed by The Centre for Applied Genomics (Hospital for Sick Children) using the Illumina pipeline where initial processing of the image files resulted in the production of the FASTA and FASTQ data files. These files were then sent to LC Sciences (Texas, USA) to perform standard data analysis using their proprietary software package, ACGT101-miR v3.5. In the analysis pipeline, adapter sequences where trimmed and sequencing reads were filtered based on quality and alignment to numerous databases and reference genomes. Briefly, low complexity and sequences that were <15 and >26 nucleotides in length were removed from the raw Illumina reads. This resulted in a list of total mappable reads for each sample. Sequences having less than 200 copies were further removed from the data. The resulting list of reads was mapped to RFam, RepBase and the *mus musculus* mRNA reference database (NCBI builder 37) to remove reads annotated as small RNAs other than miRNA. The filtered list was then compared to the miRBase database (version 16.0) to identify known miRNAs. Sequencing reads for each sample were normalized to the total number of mappable reads. The fold change was calculated as number of reads in HSV-1 samples per each miRNA over number of reads in the mock-infected sample.

### TaqMan Low Density Arrays (TLDAs) and quantitative real-time PCR (qRT-PCR) assays

To validate the next generation sequencing data, TLDAs following manufacturer’s recommendations (Life Technologies) were performed on the same RNA samples that were subject to NGS. Probes were normalized to snoRNA-U6 (U6), a small RNA molecule showing the least variation within Ct values between infected and control samples (data not shown). Data analysis was performed by calculating the fold changes between HSV-1 infected and control samples via the 2^-(ΔΔCt)^ method [32].

TaqMan miRNA assays (Life Technologies) were used to further confirm expression of select miRNAs in samples collected. For a representative whole brain expression profile, total RNA from each respective ½ of the brain were mixed in equal parts based on the quantity of RNA. A multiplex qRT-PCR analysis was performed on all samples tested as previously described [32]. Quantitative RT-PCR assays were run in duplicate and Ct values for each probe were normalized to the U6 control. Data was filtered to remove miRNAs with Ct values higher than 35 to take out low abundance miRNAs. Fold changes were calculated by the 2^-(ΔΔCt)^ method and data is represented as a mean and standard error of the mean. The expression of HSV-1 encoded hsv1-miR-H1 was detected using TaqMan miRNA assays as described above and relative abundances reported as 40-Ct or delta Ct that was normalized to U6.

### Laser capture microdissection and RNA extraction

Sagittal sections from FFPE brain tissue were used for this analysis to accurately identify the locations of heavily HSV-1 infected regions within the brain. Two serial sections were cut and processed such that one (8 μm thick section) was used for immunohistochemistry to detect HSV-1 within the tissue which served as a reference to visualize the infected regions. The subsequent section (30 μm thick) was placed on a PEN membrane slide for laser capture microdissection (LCM). Sections were processed following protocols described in the Arcturus Paradise PLUS Reagent System (Life Technologies). Briefly, sections for RNA isolation were stained using Cresyl Violet according to the manufacturer’s recommendations. Specific regions corresponding to the heavily HSV-1 positive cells were removed by LCM using the cut and capture feature. Areas ranging from approximately 43,000–172,000 μm^2^ were removed and RNA was extracted using the Arcturus^®^ Paradise^®^ Extraction and Isolation reagents as recommended by the manufacturer. A total of 2.5–4 μL of eluted RNA was used for qRT-PCR.

### *In situ* hybridization

MiR-141, miR-200a, miR-183 and miR-155 along with U6 (positive control) and a scrambled probe (negative control) within the brain sections were detected by *in situ* hybridization as previously described [33]. A total of 40 nM of the linearized double DIG-labeled miRNA-specific LNA probe (Exiqon) in ready to use hybridization solution (BioChain Institute, Inc.) was added to the slides and hybridized overnight at either 56°C for miR-141, 52°C for miR-200a, 58°C for miR-183, 49°C for miR-155, 60°C for U6 or 57°C for Scrambled probes.

To detect levels of miRNAs within neuronal population we performed a co-label with the double DIG-labeled LNA probe for miR-141 and an antibody against NeuN to detect neuronal nuclei or SMI32 to detect neurofilament H, a cytoplasmic structure present in many CNS neurons. Briefly, *in situ* hybridization protocol was performed as previously described [33] up to and including the visualization of miRNA positive staining using the NBT/BCIP solution. Subsequently, 10 minutes of 3% H_2_O_2_ was used to block endogenous peroxidase activity. Slides used only for detecting SMI32 were further blocked using rodent block (Thermo UltraVision Quanta mouse on mouse HRP Kit) for 30 minutes. All slides were rinsed in TBS/T and incubated with the respective antibodies for 1 hour at room temperature. Slides were rinsed with TBS/T and subject to secondary antibody where UltraVision Quanto mouse on mouse HRP polymer was used for SMI32 stained sections while polymer HRP anti-rabbit secondary antibody (MaxPoly-One polymer HRP rabbit detection kit DAB) was used for NeuN staining. Secondary antibody treatment lasted for 15 minutes at room temperature and the staining was visualized using DAB for 1 minute. Slides were subsequently washed in water and counterstained with Nuclear Fast Red (Vector Laboratories Inc.) for 2 minutes. Slides were then rinsed in water, air dried, clarified in xylene and mounted using Permount. Antibodies used: anti-NeuN (rabbit; 1:1000; Millipore); anti-SMI32 (mouse; 1:4000; Covance).

### Whole mouse genome transcriptomic profiling and analysis

To determine gene expression changes within viral infected brain regions, specifically the hippocampus and cerebellum, microdissection was performed on serial sections of the brain from both HSV-1 and mock-infected samples. Total RNA was extracted using the RNAqueous-Micro Kit (Life Technologies) following the manufacturers’ recommendations and total RNA was assayed on the Agilent whole mouse genome 4×44K arrays (Agilent Technologies Inc.) as previously described [33]. The microarray data files can be found in the Gene Expression Omnibus # (GSE51040).

Gene expression data was filtered using Statistical Analysis of Microarrays (SAM) software to identify differences in gene expression between HSV-1 and mock-infected samples. We considered genes that were greater than 2-fold deregulated and having a false discovery rate (FDR) of less than 1% as significantly changed.

### Bioinformatic analysis of miRNA function and network mapping

TargetScan version 6.2 (June 2012) was used to curate a list of predicted mouse specific miRNA target genes for miR-200/182 members [34]. We used Ingenuity Pathways Analysis (IPA; https://analysis.ingenuity.com), a curated database that compiles published data on gene functions and interactions, to map molecular pathways and networks populated by predicted miRNA targets. Canonical pathway and network analysis was carried out by uploading the predicted genes targeted by these upregulated miRNAs following a screen to select those genes that are also expressed at detectable levels during HSVE by microarray analysis. Networks were built based on the number of potential miRNA targets that interact with other potential targets and with other genes in the IPA database. The significance of association between genes and pathway was measured by the Fisher’s exact test p-value (p < 0.05).

### Luciferase assay

Primary mouse cortical cultures were prepared from embryonic day 18–20 CD-1 mice. Briefly, brains were collected, meninges removed and cortex was microdissected and pooled from 3–4 pups per each culture preparation. Tissues were chemically digested by incubating in papain mixture for 4 minutes at 37°C, washed three times in trypsin inhibitor and washed once in plating medium, NbActive 1 (BrainBits). Tissues were triturated in plating media and seeded into 24-well plates at 125,000 cells/well. Four days after plating, Lipofectamine 2000 (Invitrogen) was used to transfect 480 ng of the Firefly/Renilla Duo-Luciferase reporter vector containing the mouse Syndecan 2 (*Sdc2*) 3’ UTR (Genecopoeia) along with either a pre-microRNA or miRNA scrambled sequence as negative control (Life Technologies) at a final concentration of 25 nM per well. Plasmid only was also transfected for normalization purposes. Briefly, the DNA plasmid and RNA molecules were mixed with a 1:50 dilution of Lipofectamine 2000 in Neurobasal medium (Life Technologies) and incubated at room temperature for 20 minutes. This mixture was added to 400 μL of media (1:5 dilution) and cultures were incubated for 18–20 hours at 37°C. The expression of Firefly and Renilla were analyzed using the Luc-Pair DuoTM-Luciferase Assay kit (Genecopoeia) following the manufacturer’s protocol. Briefly, 20 μL of the cell lysates were used for the assay. Ratio calculated in each biological replicate was normalized to the plasmid only expression. A two tailed student’s t-test was used to assess significance.

### Statistical analysis

GraphPad Prism 6.05 was used to perform statistical analysis for the qRT-PCR data; mean ± SEM are reported for each group. Standard, one-tailed, unpaired t-test was performed; p-values with < 0.05 define significance.

## Results

### Global changes in miRNA expression during HSVE

**Fig 1:**
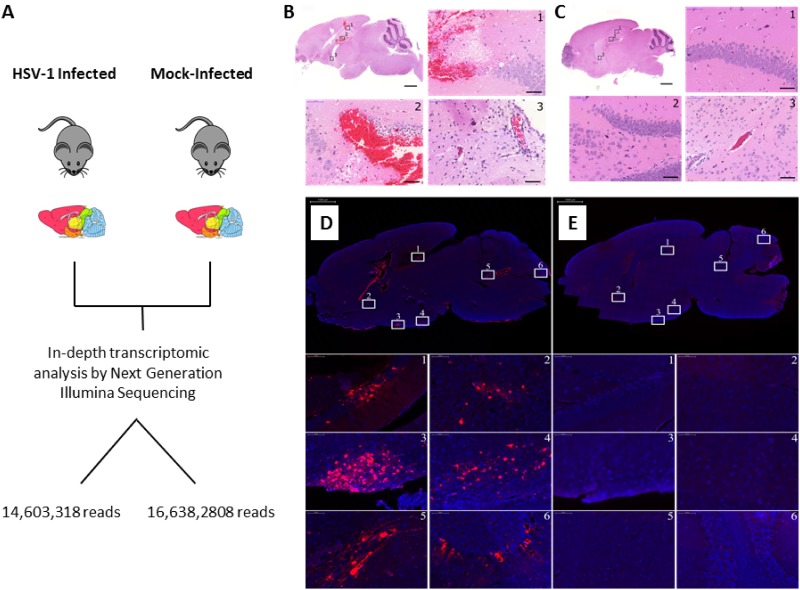
Pathology in a mouse model of acute HSVE. (A) Schematic representation of the animal experiments and process used in these studies to profile miRNAs by next generation sequencing. (B-C) Hematoxylin and Eosin staining depicting severe hemorrhaging and mononuclear cell infiltration within the blood vessels with prominent perivascular cuffing in (B) HSV-1 infected tissue. This pathogloy was not observed in (C) mock-infected mice inoculated with Vero cell suspension. Scale bars: low magnification images = 1000 μm; high magnification images = 50 μm. (D-E) Immunofluorescent images depicting HSV-1 positive cells (red) counterstained with DAPI (blue) in (D) HSV-1 and (E) mock-infected brain tissue. The magnified brain regions: (1) dentate gyrase, (2) caudate putamen, (3 and 4) hypothalamus regions, (5) pons and (6) cerebellum. Scale bars: low magnification images = 1000 μm; high magnification images = 50 μm.

To obtain an acute model of HSVE, SJL mice were intracerebrally inoculated with HSV-1 strain F (Fig 1A). All infected animals rapidly developed symptoms of encephalitis (hunched posture, ruffled fur, circling, apathy and seizures) and were sacrificed at 28 or 48 hours post-infection, along with age-matched controls. Histopathological analysis of viral infected brains showed characteristic HSV-1 lesions, congestion and dilation of blood vessels, mononuclear cell infiltration, prominent perivascular cuffing and tissue damage (Fig 1B). Brain tissue stained positive for HSV-1 by immunofluorescence, confirming the dissemination and replication of the virus (Fig 1D). HSV-1 positive areas correlated with widespread activation of astrocytes (Fig A in S1 File) and modest microgliosis that coincided with severely HSV-1 infected brain regions (Fig A in S1 File), a pathology previously documented by others [35–36]. This pathology was not detected in mock-infected brain tissue (Fig 1C and 1E)

**Fig 2:**
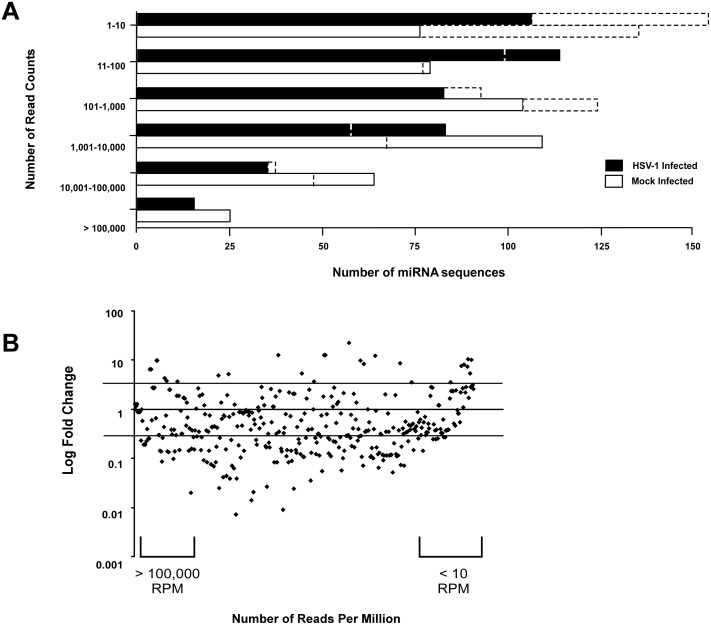
Deregulation of miRNAs during HSVE. (A) Number of miRNAs grouped based on number of read counts sequenced in HSV-1 and mock-infected samples, before normalization (dashed bars) and after normalization (solid bars) of sequencing data. (B) Mapping the fold change over the number of reads per million for each respective miRNA identified 50 upregulated and 166 downregulated miRNAs that were changed by equal to or more than 2-fold. (After filtering low abundance transcripts with less than 200 reads 24 miRNAs were upregulated and 54 downregulated) (C) The expression of HSV-1 encoded hsv1-miR-H1 was determined by the TaqMan miRNA assay in HSV-1 and mock-infected samples.

Using NGS, we profiled changes in miRNA expression during acute HSV-1 encephalitis from mouse brain tissue. Briefly, total RNA was isolated from half (left hemisphere) of the HSV-1 and mock-infected (PBS) brains from animals sacrificed 48 hours post-infection. This region contained the injection site. Small RNAs (19–26 nucleotides in length) were isolated and used to generate cDNA libraries. Libraries were sequenced and a total of 14,603,318 and 16,638,280 sequence reads were generated from the HSV-1 infected and mock-infected tissues, respectively. A series of filtering steps were subsequently performed on these data (Table A in S1 File). Comparing the number of read counts between HSV-1 and mock-infected samples revealed a global decrease in host miRNA transcripts from HSV-1 infected mice (Fig 2A). This was expected as HSV-1 shuts down global transcription during infection ([37] and has been found to inhibit RNAi machinery ([38]. Reads were normalized and low abundance transcripts were filtered out by removing miRNAs with read counts below 200. The ratio between HSV-1 and mock-infected mice were calculated to depict fold change. In total, 78 unique miRNAs were altered (24 increased and 54 decreased) more than 2.5-fold in comparison to mock-infected mice (Fig 2B; Tables B and C in S1 File). Some of the induced miRNAs included miR-146a, miR-146b and let-7g; all previously reported to be deregulated as part of an acute inflammatory response to infection [39–41]. Interestingly, 6 members of the related and often co-transcribed miRNA-200 family (miR-200a,b,c/miR-141/miR-429) and miRNA-182 cluster (miR-182/miR-183), henceforth referred to collectively as miR-200/182, were amongst the highest induced in HSV-1 infected brain. All of these miRNAs were increased between 2.5- and 22-fold. In addition the miR-429, a member of the miRNA-200 family, was upregulated 2-fold showing a similar trend of induction.

A number of HSV-1-encoded miRNAs have been shown to be expressed in viral infected cells [42]. Using qRT-PCR we assayed the expression of one of these miRNAs, hsv1-miR-H1, that is reported to be expressed in cells undergoing lytic infection. Using this technique, we were able to detect hsv1-miR-H1 in the brain of a representative HSV-1 infected mouse but not in a mock-infected sample (Fig 2C). However, we did not detect any HSV-1 encoded miRNAs by mapped our NGS reads against the HSV-1 strain F genome. We concluded that, in this case, NGS was not sensitive enough to detect hsv-1- miR-H1 in brain tissue. Viral miRNAs would only be expressed in a small proportion of brain cells harbouring replicating virus, and this represents only a small fraction of the total brain transcript population, presumably less than 1 in ~14.5 million small RNA reads detected in our analysis.

### miR-200/182 expression was upregulated during HSVE

We used a second method to assess global changes in miRNA expression, TaqMan Low Density Arrays (TLDA), to validate the expression of miRNAs that were detected by NGS (161 of the miRNAs that were detected by NGS were represented on the 384-well TLDA). We found that 17 miRNAs were similarly deregulated in TLDA (p-value ≤ 0.01) and NGS, of which 12 were upregulated and 5 downregulated in HSV-1 infected brain tissue (Table 1). However, 7 miRNAs showed an opposite expression profile between the two platforms: when NGS data indicated a decrease, TLDA platform detected an induction in fold change, and vice versa (Table D in S1 File). This discrepancy in expression profiles is likely a consequence of differences in sensitivity, specificity and linearity between the two methods, resulting in a variation in detecting some miRNAs.

**Table 1:** The list of miRNAs that were further validated by TLDA assays in HSV-1 as compared to mock-infected samples.

MiRNA	Fold Change (NGS)	Validated by TLDA
**UPREGULATED**		**(Fold Change of ≥ 2)**
mir-183	22.6	57.8
mir-141	12.1	93.8
mir-200b	9.9	36.3
mir-200c	8.6	119.5
mir-200a	5.3	32.3
mir-146b	5.2	2.4
mir-182	4.9	8.0
let-7g	4.3	2.2
let-7d	3.8	2.4
mir-148b	2.9	15.1
miR-146a	2.8	2.3
mir-186	2.5	2.6
mir-26a	2.5	2.8
**DOWNREGULATED**		**(Fold Change of ≤ -2)**
mir-125a	-10.9	-2.3
miR-132	-10.2	-2.2
mir-99a	-7.2	-2.2
mir-34a	-6.9	-2.0
mir-497	-4.3	-2.3
mir-93	-3.2	-2.4

**Fig 3:**
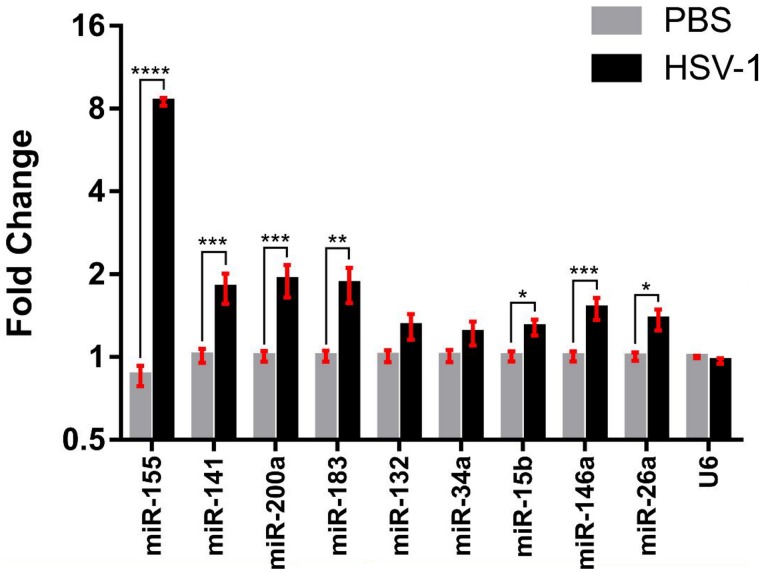
miR-200/182 and several others were upregulated in HSVE brain samples. Fold change of miRNAs compared between HSV-1 infected and PBS treated whole brain tissues. U6 served as the normalization control for all validations. Data is represented as mean ± SEM (n = 4). A one-tailed unpaired t-test was used to calculate significance where * p<0.05; ** p<0.01; *** p<0.005; **** p<0.001.

To further validate that a subset of miRNAs are induced during HSVE, we performed a second animal experiment where mice were intracerebrally inoculated with either HSV-1 or PBS (n = 4 per treatment). Brain samples were collected from these mice at the onset of clinical signs (apathy, hunched posture, ruffled fur) rather than the appearance of more the more severe clinical signs previously observed (circling and seizures). We performed qRT-PCR to validate the upregulation of 5 miRNAs identified as deregulated by NGS and TLDA miR-141, miR-200a, miR-183, miR-26a, miR-146a, miR-132, miR-34a. Additionally, we chose miR-155 and miR-15b, proinflammatory miRNAs previously shown to be upregulated in HSV-1 infection, that were filtered from our analysis due to the low abundance of their transcripts. We confirmed that 5 miRNAs identified as upregulated in the previous infection by both NGS and TLDA, were also induced in HSV-1 infected whole brain tissue from this independent animal experiment (Fig 3). In addition miR-155 and miR-15b were also induced; miR-155 with a large fold change of 8.5 (p-value < 2.3^−18^). MiR-141/200a/183 were induced by approximately 2-fold (p-value<0.01) while miR-26a was upregulated by ~1.5-fold (p-value<0.05) and miR-146a by 1.5-fold (p-value<0.005), similar to data obtained by NGS. Contrary to the NGS and TLDA data however, no significant downregulation in miR-132 and miR-34a levels was found. A potential explanation for this is that these miRNAs decreased in abundance in relation to the more advanced disease state of the mice in the previous experiment, and this was associated with more severe tissue damage.

### miR-200/182 expression was induction in HSV-1 positive areas of the brain

**Fig 4:**
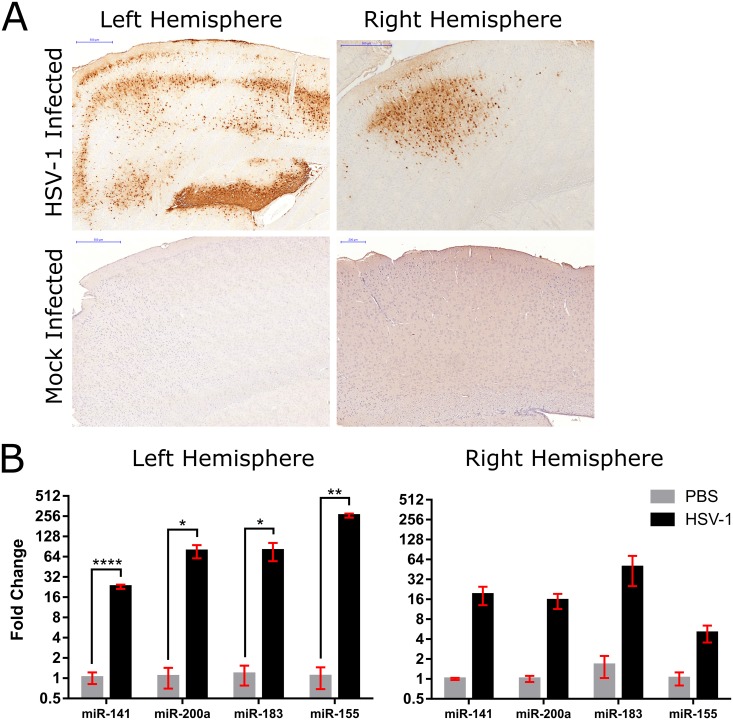
Upregulation of miR-200/182 members and miR-155 was detected in HSV-1 infected cortex region. (A) Immunohistochemistry images depicting the region of the cortex that was removed by laser capture microdissection from both left and right brain hemispheres of HSV-1 and mock-infected samples. HSV-1 positive cells are brown. Scale bar = 500 or 200 μm. (B) Fold change of miRNAs compared between HSV-1 infected and PBS treated cortical brain regions. Data is represented as mean ± SEM. A one-tailed unpaired t-test was used to calculate significance where * p<0.05; ** p<0.01; **** p<0.001.

Previous studies have shown the miR-200 family to be induced by oxidative stress and play a role in apoptosis and so we hypothesized that the significant induction of these miRNAs we saw in our model of HSV-1 induced encephalitis may be related to tissue damage during infection [43]. We therefore used laser capture microdissection (LCM) to specifically remove a region of the brain containing significant signs of infection to correlate the expression of these miRNAs with the site of disease. We chose an area within the left cortex of one infected mouse that contained a group of HSV-1 infected cells that we visualized by immunohistochemistry and H & E staining in consecutive tissue sections. A corresponding brain region from a mock-infected mouse, a control, was also removed. Additionally, the corresponding cortex region from the right hemisphere of HSV-1 infected and control mice were also processed (Fig 4A and Fig B in S1 File). Of note, HSV-1 positive cells were also identified by immunohistochemistry in the right hemisphere of the viral infected mouse; however, the number of these cells was significantly fewer when compared to the left hemisphere (the site of intracerebral inoculation), providing a qualitatively matched control. Total RNA was extracted from these samples and real-time PCR was used to profile for 3 select miR-200/182 members (miR-141, miR-200a and miR-183) as well as miR-155. The expression of all 4 host miRNAs was induced in HSV-1 infected tissue as compared to controls. Both miR-200a and miR-183 were approximately 78-fold upregulated (p-value<0.05) while miR-141 was 23-fold upregulated (p-value<0.001) (Fig 4B). MiR-155 was also found to be highly induced, reaching approximately a 260-fold upregulation (p-value<0.01). All 4 of these miRNAs were also upregulated in the right cortex of the infected brain but at a lower fold change than for the left cortex (~18-fold for miR-141, ~15-fold for miR-200a, ~49-fold for miR-183 and ~5-fold for miR-155), correlating with the relatively lower number of cells stained for HSV-1. We found that hsv1-miR-H1 expression was also lower in cells extracted from the right versus the left cortex (Fig C in S1 File), however, the viral transcript copy numbers were not determined quantitatively.

### Induction of miR-200/182 expression visualized by *in situ* hybridization in brain tissue

We used ISH to visualize the expression of a subset of induced miRNAs (miR-141, miR-183, miR-200a and miR-155) within the brain of infected mice. Increased staining in the HSV-1 versus mock-infected brain was clearly evident for miR-141, miR-183, miR-200a and miR-155 throughout the tissue (Fig D in S1 File). Additionally, we stained for the presence of U6, a non-coding snoRNA, as a positive control and found abundant and ubiquitous expression within both infected and control brain tissues. A probe designed to detect a scrambled RNA sequence was used as a negative control and showed no detectable signal in brain samples.

**Fig 5:**
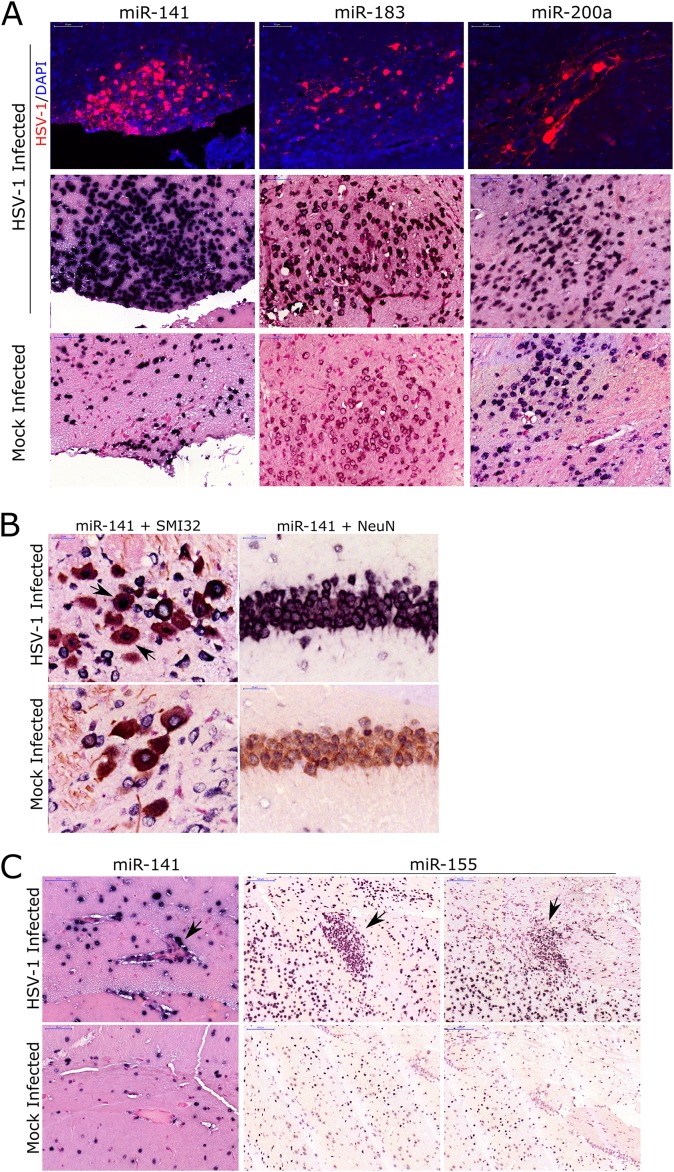
Detection of miR-200/182 upregulation in HSVE brain tissues by *in situ* hybridization. (A) Immunofluorescence images for HSV-1 (red) in HSV-1 and mock-infected brain tissue in relation to *in situ* hybridization for miR-141, miR-183 and miR-200a within similar brain areas. Hypothalamus region coinsides with miR-141 and miR-182 staining while pons region coinsides with staining for miR-200a. Scale bar = 50 μm. (B) Co-staining of neurons (brown) by an antibody against SMI32 (cerebellum) or NeuN (hippocampus) and an ISH probe against miR-141 (purple). Arrows indicate neurons that are both miR-141 and SMI32 positive in HSV-1 infected samples. Scale bar = 20 μm. (C) ISH staining (purple) for miR-141 in the hippocampus region (arrow indicates endothelial cells) and miR-155 in ventral striatum and caudate putamen regions (arrows indicate microglial modules) of HSV-1 and mock-infected brain samples. Scale bar = 100 μm.

We also compared the patterns of miRNA staining as determined by ISH to that of HSV-1 on serial tissue sections. More intense miRNA staining was detected in HSV-1 positive regions (Fig 5A), in accordance with our real-time PCR data. Particularly, we saw increased staining for miR-141 and miR-183 in the hypothalamus, a region of the brain acutely affected during HSV-1 infection [44]. Increased staining was also observed for miR-200a within the heavily HSV-1 infected pons region. Of note, several regions distal to the HSV-1 positive areas, such as parts of the cortex, also showed increased staining for these miRNAs (Fig E in S1 File).

The expression of the miR-200 family has been shown to be particularly enriched in epithelial and endothelial tissues [43, 45]. Based on the staining patterns of miR-200/182 as determined by ISH, it appeared that members of these miRNA families could also be expressed and induced in neurons. We confirmed the expression of miR-141 in some neuronal subtypes by co-labelling with either SMI32, an antibody that binds to neurofilament H and allows visualization of neuronal cell bodies and dendrites of many central nervous system neurons, or NeuN, a nuclear marker for neurons. We found pronounced miR-141 staining within certain population of neurons in HSVE infected tissue (Fig 5B). MiR-141 also showed particularly intense staining in endothelial cells of blood vessels as expected based on previous studies (Fig 5C). Increased expression of miR-155 was detected throughout the brain with staining in some areas showing patterns characteristic of infiltrating inflammatory cells (Fig 5C).

### Bioinformatic analysis of miR-200/182 function identifies a potential role in heparan sulfate proteoglycan (HSPG) biosynthesis

**Fig 6:**
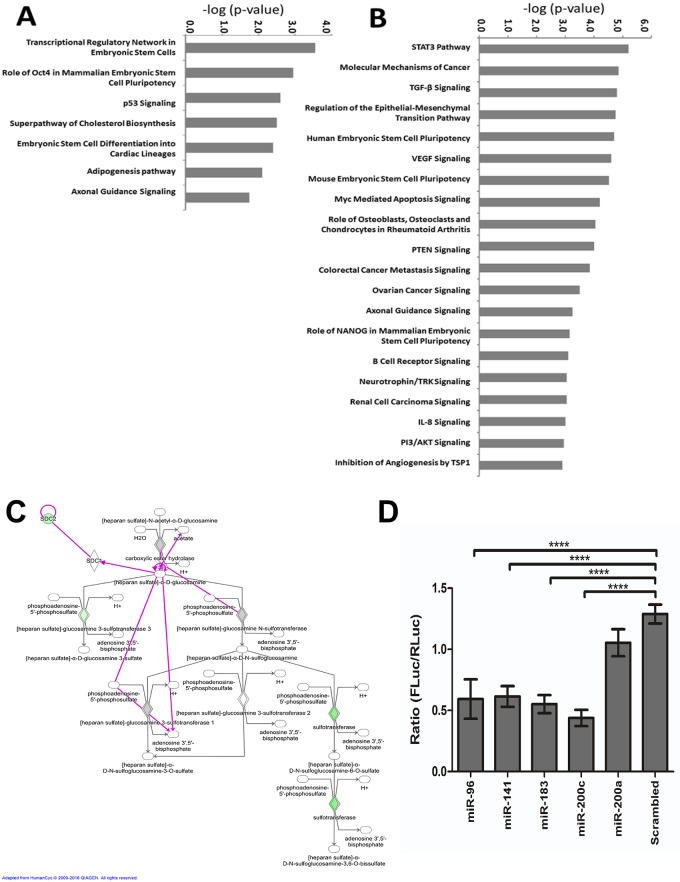
miR-200/182 members may regulate expression of heparan sulfate proteoglycan, syndecan-2 (*Sdc2*). (A) Top 7 canonical pathways enriched (p < 0.05) by downregulated genes potentially targeted by miR-200/182 miRNAs. (B) Top 20 canonical pathways enriched (p < 0.05) by downregulated genes potentially targeted by miR-155, miR-146a, miR-146b and miR-15b. (C) Ingenuity Pathway Analysis generated diagram to show the biosynthetic pathway of HSPGs. In green are the gene targets of miR200/182 members that are down-regulated in brain tissue acutely infected with HSV-1. In grey are putative targets of miR200/182 members whose expression was determined to be unchanged. (D) Luciferase reporter assay depicting the miRNAs which were found to decrease expression of the mouse *Sdc2* 3’ UTR as compared to a scrambled miRNA sequence as control. Data was normalized to *Renilla* luciferase followed by plasmid only levels across replicates. Data is represented as mean ± SEM (n = 2). A two-tailed t-test was used to calculate significance where **** p<0.001.

Given the upregulation of miR-200/182 members during acute HSV-1 infection, we employed a bioinformatics approach to explore their functionality within the context of HSV-1-induced encephalitis. First, the potential mRNA targets for these miRNAs were predicted using TargetScan 6.2 and a conservation score cut-off of 0.2 was employed. The conservation score, or P_CT_, estimates the probability of conserved targeting [46] and was shown to be effective when correlating increased miRNA binding with decreased target gene expression [47]. Considering that miRNAs and their targets can only interact within the same tissues and cells that express them concurrently, the potential miRNA target list was further trimmed based on mRNAs detected in HSV-1 infected brain tissue. From a list of 18,756 mouse genes that were detected using microarrays in HSV-1 infected mouse brains, 2137 were deregulated over 2-fold versus mock-infected controls; 982 were upregulated and 1155 were downregulated. Amongst these genes, 2260 were also predicted targets of miR-200/182, from which 200 were downregulated and 54 upregulated more than 2-fold in mouse brain following infection. A higher proportion of downregulated genes (4-fold enrichment) confirmed a significant correlation between increased miRNA binding potential and decreased target gene expression. The 200 downregulated genes were used for functional pathway and network enrichment analysis using the IPA tool. Pathway analysis revealed a statistically significant enrichment (p-value<0.05) amongst these genes for biological functions involved in cellular and tissue development, cell death and survival, cell viability and proliferation and angiogenesis. The 7 canonical pathways that were significantly enriched (p-value<0.05) in the group of genes that were downregulated in HSVE and also predicted targets of miR-200/182 are shown in Fig 6A. In contrast, the most significant canonical pathways reflecting the 180 genes predicted to be downregulated by the inflammation related miRNAs (miR-155, miR-146a, miR-146b and miR-15b) are primarily involved in differentiation of myeloid cells and organismal injury. The top 20 of the 97 canonical pathways statistically enriched (p-value<0.05) are shown in Fig 6B.

A number of downregulated genes targeted by miR-200/182 (including the sulfotransferases *HS3ST1*, *HS3ST3A1*, *HS6ST2* and *SDC2*) are involved in heparan sulfate proteoglycan (HSPG) biosynthesis (Fig 6C). This was of particular interest as HSPGs play important roles in the HSV-1 life-cycle and can be exploitated by the virus as attachment receptors for viral entry [48]. One heparan sulfate proteoglycan, *Sdc2*, was predicted by TargetScan to be targeted at multiple conserved sites by at least 5 different members of the miR-200/182 miRNA group; miR-141, miR-200b/c, miR-182, miR-96 and miR-183. To validate this interaction experimentally, we used a luciferase reporter assay containing the *Sdc2* 3′ UTR cloned downstream of the firefly luciferase gene. We confirmed that miR-96, miR-141, miR-183 and miR-200c all bound to the *Sdc2* 3’UTR, resulting in ~2-fold downregulation (p-value<0.001) of the luciferase reporter gene (Fig 6D).

## Discussion

We examined the changes in cellular miRNA expression in acute HSVE mouse brain tissues and using NGS, a list of miRNAs that were deregulated during HSVE was identified. We further validated the significant induction of several miRNAs within HSV-1 infected brain and found that the expression of these miRNAs was associated with heavily HSV-1 infected regions. We were especially interested in miRNAs that were upregulated in HSVE because HSV-1 shuts down global transcription during infection (36) and can inhibit RNAi machinery (37). Therefore, it is possible that upregulated miRNAs would more likely play a role during pathogenicity of HSVE.

Notably, miR-146a, miR-15b and miR-155 that are known to be induced as part of inflammatory pathways in response to numerous stimuli, including viral infection [39, 41, 49–56], were significantly upregulated in the brains of HSV-1 infected animals during acute clinical disease. However, antagonistic roles have been found between these miRNAs; while miR-146a functions to inhibit inflammation, miR-15b and miR-155 are proinflammatory. MiR-146a suppresses the expression of NF-κB activity and disruption of the Jak-STAT signaling pathway [57]. In Japanese encephalitis virus (JEV) infection of microglial cells, miR-146a was upregulated and helped the virus evade the host immune response [51]. During HSVE however, the rampant stimulation of an immune response becomes deleterious to the brain tissue, causing excessive damage to bystander cells not infected by HSV-1 [58]. Perhaps upregulation of miR-146a in HSVE brain serves to dampen the severity of the immune response to prevent excessive damage to the brain. In turn, miR-15b was found to inhibit a suppressor of RIG-I, resulting in higher production of proinflammatory cytokines [49]. Upregulation of this miRNA was detected in JEV infection of mouse brain and inhibition of miR-15b expression resulted in decreased tissue damage of the brain, decreased viral burden and improved survival of mice [49]. Although the functional role of miR-15b has not been identified in HSVE, it may also stimulate proinflammatory responses.

Out of the 3 immune related miRNAs, miR-155 was upregulated the most (>8-fold) in HSV-1 whole brain tissue. This miRNA is enriched in hematopoietic cells including B-cells, T-cells, monocytes and granulocytes [59]. When we investigated the spatial expression of miR-155 within HSV-1 infected brain tissue we found staining largely in small cells with a microglial phenotype as well as in cells concentrated around blood vessels and in microglial nodules, suggesting that miR-155 may be upregulated in immune cells that are infiltrating into the brain. The stimulation of a pro-inflammatory response in microglia is known to be mediated, at least in part, by the upregulation of miR-155 [60]. Furthermore, over-expression of miR-155 in microglial cells was recently found to negatively regulate JEV replication and thereby reduce viral-induced gene expression in host cells [56]. Interestingly, we found miR-155 to be ~260-fold upregulated in areas strongly positive for HSV-1 infected cells suggests that this miRNA may be similarly inhibiting the progression of viral pathogenicity. In fact, miR-155 knock-out mice are more susceptible to HSV replication and dissemination in the nervous system [30]. Mechanistically, a deficiency in CD8+ T-cells similarly to that described in lymphocytic choriomeningitis virus and influenza infection may be responsible [55, 61–62]. We also found enhanced miR-155 expression in endothelial cells. Recent evidence suggests that miR-155 stimulates a cytokine-induced increase in permeability of the blood-brain barrier during inflammation [63] also contributing to the exacerbation of the inflammatory response during HSVE. Another cell-type of interest as a modulator of neuroinflammation, as well as eliciting neuroprotective responses in diseased brain are astrocytes. We showed in Fig A in S1 File that HSVE in our mouse model was associated with astrocytosis and the role of astrocytes in infectious disease in the brain is a relatively unexplored avenue for further research. MiR-146a and miR-155 have both been shown to play roles in astrocyte-mediated neuroinflammation [64]. In addition miR-141 is expressed in normal human astrocytes and the role for this miRNA in these cells, and other members of miR-200 family and miR-182 cluster, has not yet been investigated [65].

The induction of multiple members of the highly related, and often co-transcribed, miRNA-200 family and miRNA-182 cluster was our most striking finding. The upregulation of these miRNAs was associated with regions of the brain that were positive for HSV-1 infected cells. *In situ* hybridization for miR-141, miR-200a and miR-183 further showed that the increased expression was not limited to cells with a glial or lymphocyte phenotype but also included neurons and likely other resident cell-types of the central nervous system (CNS) such as microglia, astrocytes and endothelial cells. This observation suggests that these miRNAs are involved in a more generalized host response in regions where HSV-1 replication was occurring in the brain tissue. We appreciate that further work to pin-point the deregulation of these miRNAs in specific CNS cell sub-types would be required to confirm this hypothesis. These miRNAs have not been linked to host immunity to infection and inflammation in the same way as miR-155. However, a report describing the targeting of Myd88 by miR-200b and miR-200c followed by subsequent downregulation of proinflammatory molecules in THP-1 cells exist but is not a common theme in the literature [66]. There are, however, reports listing the deregulation of miR-200/182 members in a number of other viral disease models. In particular, several miR-200/182 members were downregulated in rabies virus infection [67] and West Nile virus infection [16] of mice while upregulated in HCMV [15,68] and influenza infection of cell culture [69–70]. Clearly, these miRNAs are collectively co-expressed during numerous viral infections, which suggest their involvement in a host response mechanism to viral disease pathogenesis. However, the function of these miRNAs within the context of HSV-1 pathogenicity remains unknown.

Using the target prediction software TargetScan in conjunction with gene expression profiling, we found that miR-200/182 may in fact modulate the biosynthesis of heparan sulfate proteoglycans (HSPGs). It is well-known that HSPGs are critical for the cellular attachment of HSV-1 as well as many other viruses, including flavivirus, adenovirus, papillomavirus and retroviruses [45, 71]. They are expressed on the surfaces of most mammalian cells and consist of a core protein with O-linked heparan sulfate polysaccharide chains. The HSPG core proteins include the membrane-spanning syndecans (SDCs), the lycosylphosphatidylinositol-linked glypicans (GPCs), the basement membrane proteoglycan perlecan (HSPG2), and agrin (AGRN). In the case of HSV-1, HSPGs also induce fusion of the virus with the cell membrane, either at the surface or during endocytosis or phagocytosis [72–74]. It was recently shown that knock-down of the HSPG core proteins SDC1 and SDC2 reduced HSV-1 entry, plaque formation and increased survival of infected cells [31]. HSV-1 infection was also shown to increase SDC1 and SDC2 protein synthesis and total cell surface expression of heparan sulfate [31]. Using luciferase assays, we found that miR-96, miR-141, miR-183 and miR-200c all caused a downregulation of *Sdc2* expression. The induction of miR-200/182 miRNAs in HSV-1 infected brains may therefore play a role as a host mechanism to mitigate virus entry and spread by downregulating SDC2.

## Conclusions

Herein, we identify alterations in miRNA expression in the brain in response to acute encephalitis caused by infection with HSV-1. We identified several immune-related miRNAs that were induced in HSVE affected brain tissue, including proinflammatory miR-155 that was previously shown to play a role in the host response to HSV-1 infection by preventing the replication and dissemination of HSV-1 within the brain. Furthermore, we identified the co-ordinate dysregulation of miR-200/182 family members during acute encephalitis. These miRNAs were induced in areas of the tissue heavily infected by HSV-1. In addition, a potential role for miR-200/182 members is the regulation of HSPG synthesis. Several miR-200/182 members are able to downregulate the expression of *Sdc2*, a core protein of HSPGs that is important for attachment of numerous viruses, including HSV-1, for entry into the host cell. Further understanding of the role of these miRNAs during HSV-1 infection is required to determine whether modulation of their expression could have potential therapeutic value in acute HSV-1.

## Supporting Information

S1 File Containing Figs A-E and Tables A-D. (DOCX)
