# Correction: Induction of Multiple miR-200/182 Members in the Brains of Mice Are Associated with Acute Herpes Simplex Virus 1 Encephalitis

**DOI:** 10.1371/journal.pone.0172815

**Published:** 2017-02-21

**Authors:** Anna Majer, Kyle A. Caligiuri, Kamilla K. Gale, Yulian Niu, Clark S. Phillipson, Timothy F. Booth, Stephanie A. Booth

There are two errors in the “miR-200/182 expression was induction in HSV-1 positive areas of the brain” section of the Results. “Fig B in S1 File” should be listed as “Fig D in S1 File” and “Fig C in S1 File” should be listed as “Fig E in S1 File”.

There are two errors in the “Induction of miR-200/182 expression visualized by *in situ* hybridization in brain tissue” section of the Results. In the first paragraph, “Fig D in S1 File” should be listed as “Fig B in S1 File”. In the second paragraph, “Fig E in S1 File” should be listed as “Fig C in S1 File”.

Panel C in Fig 2 does not appear. Please view the correct Fig 2 here.

**Fig 2:**
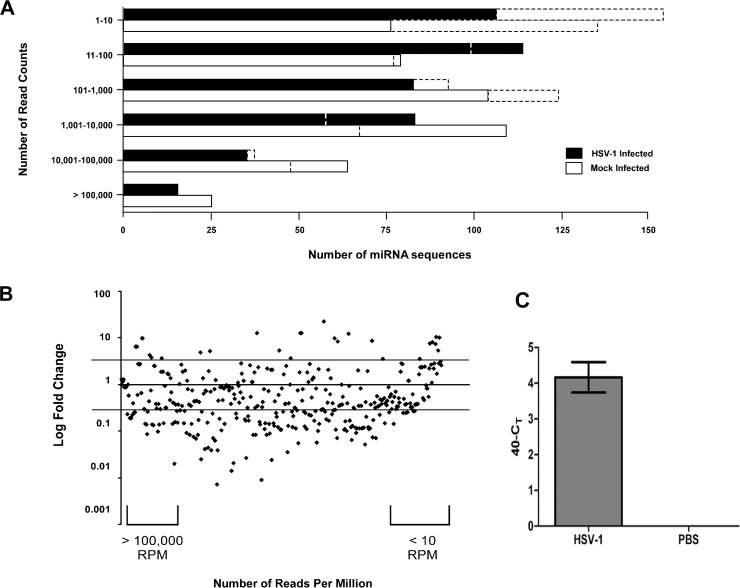
Deregulation of miRNAs during HSVE. (A) Number of miRNAs grouped based on number of read counts sequenced in HSV-1 and mock-infected samples, before normalization (dashed bars) and after normalization (solid bars) of sequencing data. (B) Mapping the fold change over the number of reads per million for each respective miRNA identified 50 upregulated and 166 downregulated miRNAs that were changed by equal to or more than 2-fold. (After filtering low abundance transcripts with less than 200 reads 24 miRNAs were upregulated and 54 downregulated) (C) The expression of HSV-1 encoded hsv1-miR-H1 was determined by the TaqMan miRNA assay in HSV-1 and mock-infected samples.
