# Supplementary material for: Induction of Multiple miR-200/182 Members in the Brains of Mice Are Associated with Acute Herpes Simplex Virus 1 Encephalitis
Source: PLoS One. 2017 Jan 3;12(1):e0169081. doi: 10.1371/journal.pone.0169081 (PMC5207681; doi:10.1371/journal.pone.0169081)
Supplement: S1 File — (DOCX) [file pone.0169081.s001.docx]

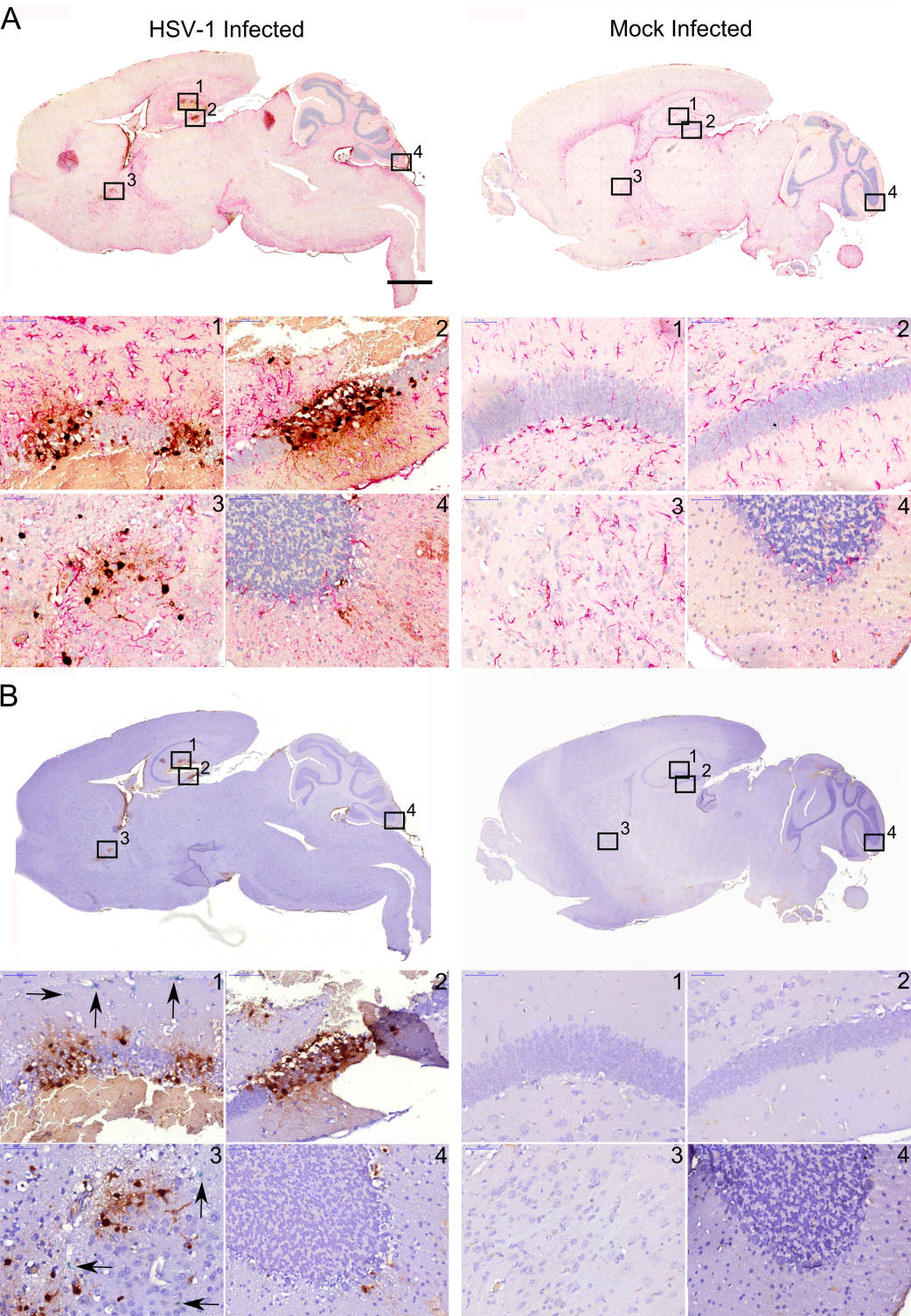


**Fig A. The degree of astrocyte and microglial spread within HSV-1 infected brain.** (**A**) Extensive astrocytosis was apparent in 4 HSV-1 infected brain regions when compared to mock-infected brain. Astrocytes are represented by the pink staining while HSV-1 infected cells are brown. (**B**) Modest microgliosis was detected in HSVE as compared to mock-infected samples. Microgliosis is represented by the light green staining (highlighted by arrows) and the presence of HSV-1 is depicted by the brown staining. Areas 1 and 2 correspond to dentate gyrase, area 3 represents caudate putamen, and area 4 is found within the cerebellum. Scale bars: low magnification images = 1000 µm; high magnification images = 50 µm.

**Table A.** Summary of the generated read counts and filtering steps used in the analysis of HSV-1 and mock-infected mice.

| Description | HSV-1 Infected | Mock-Infected |
| --- | --- | --- |
| Raw Illumina Reads | **14,603,318** | 16,638,280 |
| Remove low complexity reads | - 47,595 | - 50,409 |
| Remove sequences < 15 and > 26 | - 9,525,693 | - 8,341,850 |
| Total Mappable Reads | **5,030,030** | 8,246,021 |
| Remove sequences with a copy number < 3 | -1,051,532 | - 950,631 |
| Remove sequences mapped to other databases (eg. mRNA and RFam) | - 112,512 | - 176,825 |
| Total Reads Mappable to miRNA | **3,865,986** | 7,118,565 |
| Total unique sequences mappable to miRNAs | 41,532 | 103,390 |
| Total unique miRNAs | 705 | 798 |
| Known miRNAs | 261 | 382 |

**Table B**. The list of 24 miRNAs that were increased by at least 2.5-fold in HSV-1 infected samples as compared to controls.

|  | **HSV-1 Infected** | | **Mock-Infected** | |  |
| --- | --- | --- | --- | --- | --- |
| **MiRNA** | **Raw Reads** | **RPM** | **Raw Reads** | **RPM** | **Fold Change** |
| mir-183 | 6238 | 2746.9 | 566 | 121.4 | 22.6 |
| mir-2142 | 13846 | 6097.1 | 2253 | 483.2 | 12.6 |
| mir-1 | 5456 | 2402.5 | 889 | 190.7 | 12.6 |
| mir-141 | 1633 | 719.1 | 277 | 59.4 | 12.1 |
| let-7f | 565443 | 248992.7 | 117375 | 25173.6 | 9.9 |
| mir-200b | 1742 | 767.1 | 360 | 77.2 | 9.9 |
| mir-200c | 738 | 325 | 177 | 38 | 8.6 |
| mir-96 | 1399 | 616 | 339 | 72.7 | 8.5 |
| let-7a | 667807 | 294068.7 | 212183 | 45507.1 | 6.5 |
| mir-200a | 3755 | 1653.5 | 1455 | 312.1 | 5.3 |
| mir-146b | 16911 | 7446.8 | 6616 | 1418.9 | 5.2 |
| mir-182 | 21449 | 9445.1 | 9016 | 1933.7 | 4.9 |
| let-7g | 115253 | 50751.6 | 55675 | 11940.7 | 4.3 |
| let-7d | 97569 | 42964.5 | 53154 | 11400 | 3.8 |
| let-7e | 83789 | 36896.5 | 47131 | 10108.2 | 3.7 |
| mir-144 | 2038 | 897.4 | 1134 | 243.2 | 3.7 |
| mir-148b | 4532 | 1995.7 | 3245 | 696 | 2.9 |
| let-7c | 203474 | 89599.7 | 151646 | 32523.7 | 2.8 |
| mir-146a | 1021 | 449.6 | 757 | 162.4 | 2.8 |
| let-7b | 198268 | 87307.3 | 152271 | 32657.7 | 2.7 |
| mir-21 | 31717 | 13966.6 | 24370 | 5226.7 | 2.7 |
| mir-383 | 3226 | 1420.6 | 2587 | 554.8 | 2.6 |
| mir-26a | 62034 | 27316.7 | 51458 | 11036.3 | 2.5 |
| mir-186 | 1555 | 684.7 | 1287 | 276 | 2.5 |

**Table C**. The list of 54 miRNAs that were decreased by at least 2.5-fold in HSV-1 infected samples as compared to controls.

|  | **HSV-1 Infected** | | **Mock-Infected** | |  |
| --- | --- | --- | --- | --- | --- |
| **MiRNA** | **Raw Reads** | **RPM** | **Raw Reads** | **RPM** | **Fold Change** |
| mir-328 | 284 | 125.1 | 29756 | 6381.8 | -51 |
| mir-154 | 388 | 170.9 | 11424 | 2450.1 | -14.3 |
| mir-219 | 429 | 188.9 | 11717 | 2513 | -13.3 |
| mir-211 | 440 | 193.8 | 11003 | 2359.8 | -12.2 |
| mir-204 | 560 | 246.6 | 13789 | 2957.3 | -12 |
| mir-99b | 2860 | 1259.4 | 69350 | 14873.6 | -11.8 |
| mir-125a | 816 | 359.3 | 18258 | 3915.8 | -10.9 |
| mir-132 | 2357 | 1037.9 | 49967 | 10716.5 | -10.3 |
| mir-32 | 240 | 105.7 | 4793 | 1028 | -9.7 |
| mir-125b | 3336 | 1469 | 51657 | 11078.9 | -7.5 |
| mir-99a | 3335 | 1468.6 | 49434 | 10602.2 | -7.2 |
| mir-218 | 5007 | 2204.8 | 72240 | 15493.4 | -7 |
| mir-23b | 2163 | 952.5 | 31223 | 6696.4 | -7 |
| mir-341 | 316 | 139.2 | 4525 | 970.5 | -7 |
| mir-34a | 947 | 417 | 13414 | 2876.9 | -6.9 |
| mir-100 | 3186 | 1403 | 44593 | 9563.9 | -6.8 |
| mir-23a | 1449 | 638.1 | 20156 | 4322.9 | -6.8 |
| mir-582 | 527 | 232.1 | 7038 | 1509.4 | -6.5 |
| mir-129 | 2216 | 975.8 | 29225 | 6267.9 | -6.4 |
| mir-532 | 210 | 92.5 | 2589 | 555.3 | -6 |
| mir-329 | 248 | 109.2 | 2997 | 642.8 | -5.9 |
| mir-139 | 862 | 379.6 | 10286 | 2206.1 | -5.8 |
| mir-495 | 268 | 118 | 3095 | 663.8 | -5.6 |
| mir-145 | 449 | 197.7 | 5074 | 1088.2 | -5.5 |
| mir-124 | 23918 | 10532.3 | 261594 | 56104.4 | -5.3 |
| mir-212 | 354 | 155.9 | 3831 | 821.6 | -5.3 |
| mir-299 | 282 | 124.2 | 2782 | 596.7 | -4.8 |
| mir-29c | 28164 | 12402 | 258252 | 55387.6 | -4.5 |
| mir-29a | 29981 | 13202.1 | 268998 | 57692.3 | -4.4 |
| mir-497 | 441 | 194.2 | 3855 | 826.8 | -4.3 |
| mir-330 | 653 | 287.5 | 5621 | 1205.5 | -4.2 |
| mir-30c | 28142 | 12392.3 | 225784 | 48424.2 | -3.9 |
| mir-30b | 8134 | 3581.8 | 63319 | 13580.1 | -3.8 |
| mir-598 | 1080 | 475.6 | 8366 | 1794.3 | -3.8 |
| mir-191 | 3801 | 1673.8 | 28692 | 6153.6 | -3.7 |
| mir-192 | 2634 | 1159.9 | 20070 | 4304.4 | -3.7 |
| mir-451 | 3268 | 1439.1 | 24442 | 5242.1 | -3.6 |
| mir-29b | 11819 | 5204.5 | 82133 | 17615.2 | -3.4 |
| mir-128 | 7024 | 3093 | 48739 | 10453.1 | -3.4 |
| mir-16-2 | 5753 | 2533.3 | 37877 | 8123.5 | -3.2 |
| mir-93 | 432 | 190.2 | 2800 | 600.5 | -3.2 |
| mir-496 | 358 | 157.6 | 2288 | 490.7 | -3.1 |
| mir-301b | 326 | 143.6 | 2024 | 434.1 | -3 |
| mir-27a | 3601 | 1585.7 | 21573 | 4626.8 | -2.9 |
| mir-301a | 379 | 166.9 | 2249 | 482.3 | -2.9 |
| mir-27b | 5133 | 2260.3 | 28849 | 6187.3 | -2.7 |
| mir-136 | 4456 | 1962.2 | 24568 | 5269.1 | -2.7 |
| mir-195 | 2752 | 1211.8 | 15406 | 3304.1 | -2.7 |
| mir-181a | 9273 | 4083.4 | 49666 | 10651.9 | -2.6 |
| mir-425 | 784 | 345.2 | 4252 | 911.9 | -2.6 |
| mir-19b | 250 | 110.1 | 1344 | 288.2 | -2.6 |
| mir-378 | 48280 | 21260.1 | 252603 | 54176.1 | -2.5 |
| mir-153 | 5953 | 2621.4 | 30431 | 6526.6 | -2.5 |
| mir-19a | 227 | 100 | 1168 | 250.5 | -2.5 |

**Table D.** A list of 7 miRNAs that were identified to have different expression profiles in NGS and TLDA analyses.

|  | **HSV-1 Infected** | | **Mock-Infected** | |  |  |
| --- | --- | --- | --- | --- | --- | --- |
| **MiRNA** | **Raw Reads** | **RPM** | **Raw Reads** | **RPM** | **Fold Change*** | **Fold change by TLDA** |
| mir-99b | 2860 | 1259.4 | 69350 | 14873.6 | -11.8 | 2.5 |
| mir-195 | 2752 | 1211.8 | 15406 | 3304.1 | -2.7 | 3.5 |
| mir-192 | 2634 | 1159.9 | 20070 | 4304.4 | -3.7 | 4.3 |
| mir-27a | 3601 | 1585.7 | 21573 | 4626.8 | -2.9 | 5.2 |
| mir-431 | 139 | 61.2 | 1096 | 235.1 | -3.8 | 6 |
| mir-425 | 784 | 345.2 | 4252 | 911.9 | -2.6 | 6.7 |
| mir-451 | 3268 | 1439.1 | 24442 | 5242.1 | -3.6 | 10.5 |

* fold change as calculated from next generation sequencing (NGS) data


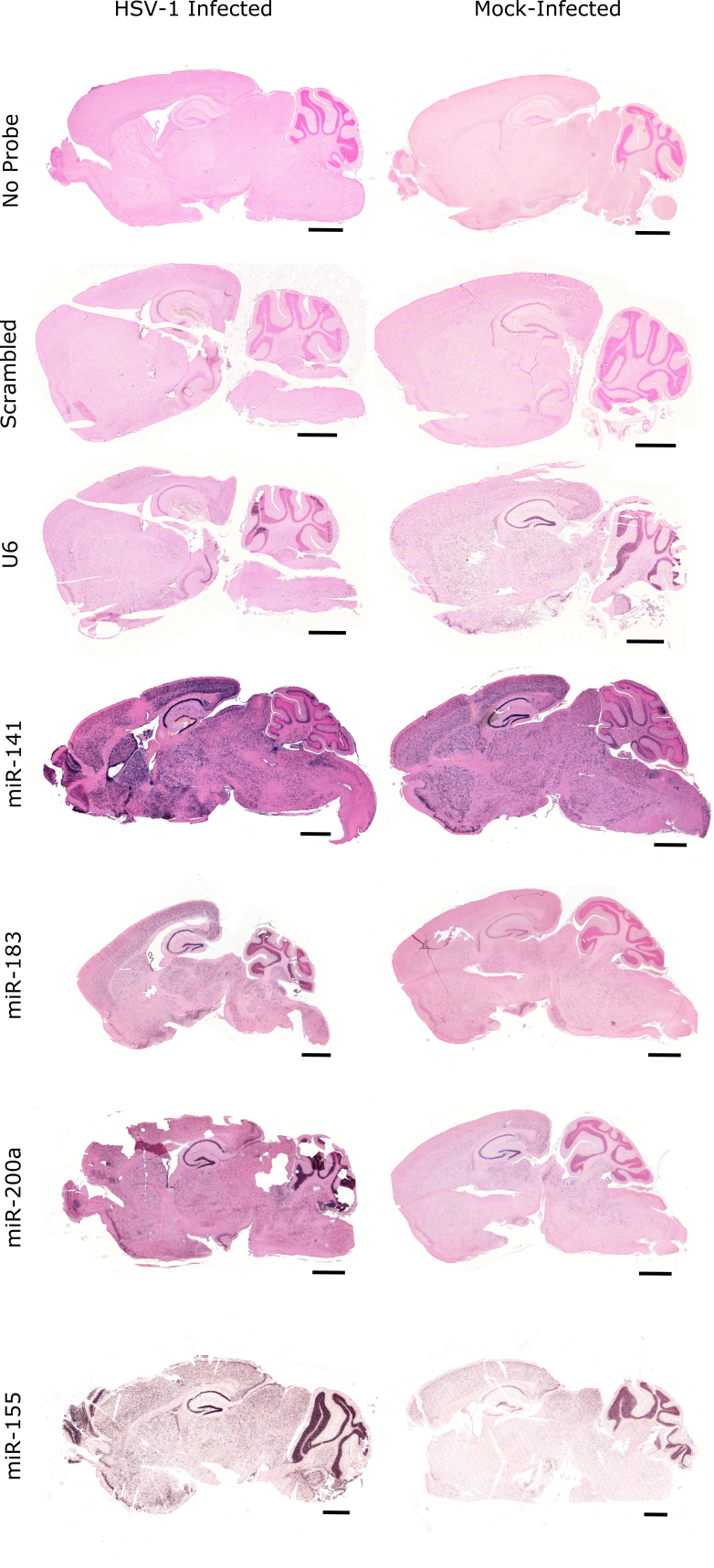


**Fig B. *In situ* hybridization for several miRNAs in HSV-1 and mock-infected brain tissue.** Samples were analyzed for staining of miR-141, miR-183 miR-200a, snoRNA U6 (positive control), scrambled (negative control) and no probe. Dark blue staining indicates more pronounced positive staining for the respective miRNA. Scale bar = 1000 µm.


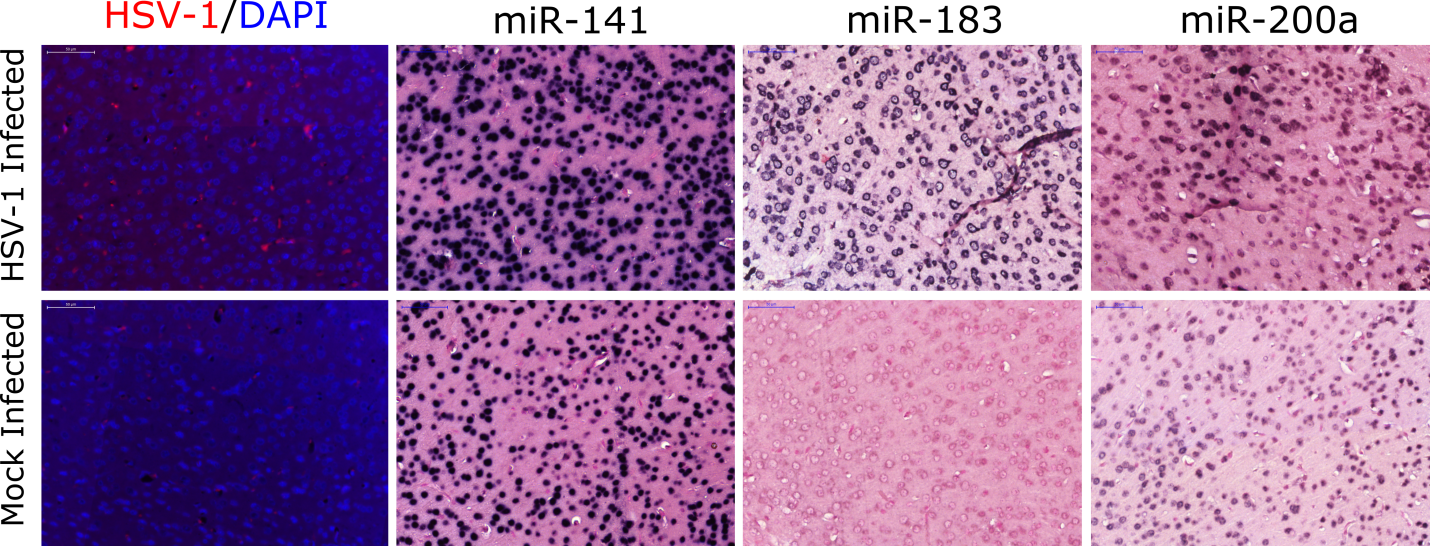


**Fig C. *In situ* hybridization of miR-141, miR-183 and miR-200a in the cortex areas not positive for HSV-1 infected cells**. Area within the cortex with minimum HSV-1 presence showed positive staining for miR-141, miR-183 and miR-200a in HSV-1 infected tissue as compared to mock-infected tissue. Immunofluorescence images represent HSV-1 infected cells (red) as compared to DAPI counterstained cell nuclei (blue). *In situ* hybridization images indicate dark purple staining for miRNA against a pink cytoplasmic counterstain. Scale bar = 50 µm.

**
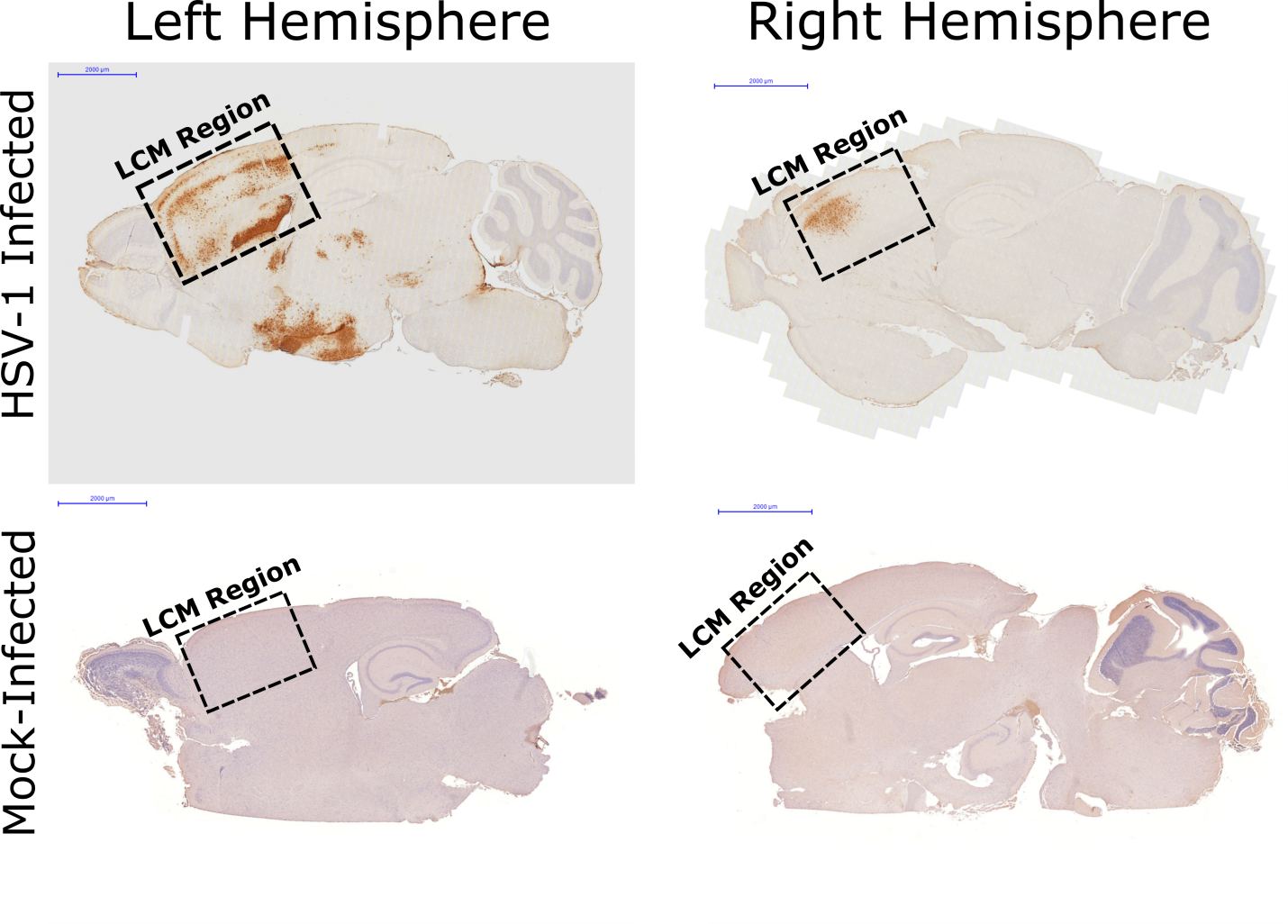
**

**Fig D. Cortical area that was removed from HSV-1 and mock-infected samples by laser capture microdissection for miRNA profiling.** The area is highlighted in each figure. HSV-1 positive staining is depicted in brown and sections were counterstained with hematoxylin. Scale bar = 2000 µm.

**Fig E. The expression of hsv1-miR-H1 as determined by TaqMan miRNA assays in the left and right cortex regions isolated by LCM.**
